# Ambient occlusion and PCV (portion de ciel visible): A new dental topographic metric and proxy of morphological wear resistance

**DOI:** 10.1371/journal.pone.0215436

**Published:** 2019-05-01

**Authors:** Michael A. Berthaume, Julia Winchester, Kornelius Kupczik

**Affiliations:** 1 Department of Bioengineering, Imperial College London, London, United Kingdom; 2 Max Planck Weizmann Center for Integrative Archaeology and Anthropology, Max Planck Institute for Evolutionary Anthropology, Leipzig, Saxony, Germany; 3 Department of Anthropology, Durham University, Durham, United Kingdom; 4 Department of Evolutionary Anthropology, Duke University, Durham, North Carolina, United States of America; Monash University, AUSTRALIA

## Abstract

Recently, ambient occlusion, quantified through portion de ciel visible (PCV) was introduced as a method for quantifying dental morphological wear resistance and reconstructing diet in mammals. Despite being used to reconstruct diet and investigate the relationship between dental form and function, no rigorous analysis has investigated the correlation between PCV and diet. Using a sample of platyrrhine and prosimians M_2_s, we show average PCV was significantly different between most dietary groups. In prosimian, insectivores had the lowest PCV, followed by folivores, omnivores, frugivores, and finally hard-object feeders. In platyrrhines, omnivores had the lowest average PCV, followed by folivores, frugivores, and finally hard-object feeders. PCV was correlated to two topographic variables (Dirichlet normal energy, DNE, and relief index, RFI) but uncorrelated to three others (orientation patch count rotated, OPCR, tooth surface area, and tooth size). The OPCR values here differed greatly from previously published values using the same sample, showing how differences in data acquisition (i.e., using 2.5D vs. 3D surfaces) can lead to drastic differences in results. Compared to other popular topographic variables, PCV performed as well or better at predicting diet in these groups, and when combined with a metric for size, the percent of successful dietary classifications reached 90%. Further, using an ontogenetic series of hominin (*Paranthropus robustus*) M_2_s, we show that PCV correlates well with probability of wear, with PCV values being higher on the portions of the occlusal surface that experience more wear (e.g., cusps and crest tips, wear facets) than the portions of the tooth that experience less. This relationship is strongest once wear facets have begun to form on the occlusal surface. These results highlight the usefulness of PCV in quantifying morphological wear resistance and predicting diet in mammals.

## Introduction

Dental topography has become a popular method for inferring diet from tooth shape in mammals [1,2]. While methods for quantifying tooth shape in a dietary manner have existed for decades (e.g. shearing quotient and ratio [3–8]), it wasn’t until the development of advanced scanning techniques and homology-free metrics that species with dissimilar occlusal morphologies could be directly compared in large scale analyses [9,10].

Some of the most popular dental topographic metrics today are the relief index (RFI), orientation patch count (OPC)/orientation patch count rotated (OPCR), and Dirichlet normal surface energy (DNE). RFI is defined as either the 3D surface area of a tooth divided by its 2D cross-sectional area, or the natural log of that ratio, and is a measure of surface relief [11–14]. While RFI is efficient at differentiating mammal diets within certain groups and clades [15], it seems to measure degree of hypsodonty, which can be problematic when attempting to quantify occlusal shape. Furthermore, tooth surface area reduces during an individual’s lifetime due to wear, but cross-sectional area does not, causing RFI to decrease with age [16,17]. This makes it difficult to gain correct dietary signatures from worn teeth when using RFI.

Orientation patch count and orientation patch count rotated (a form of OPC which normalizes for tooth orientation) are measures of occlusal complexity, and can be efficient at differentiating mammal teeth into dietary categories if there is large variation in dental complexity within the sample [9,18]. Finally, DNE is a measure of the curviness of the surface of the tooth, and is highly correlated to RFI in unworn teeth [15,18,19]. It should be noted DNE makes no distinction about the location of the curves, meaning both concave and convex curves will give the same DNE value (see Fig 3 in Bunn et al., 2011). As a result, DNE has strong signal reflecting enamel crenulations, as crenulated teeth are filled with concave curves [20–22].

**Fig 1:**
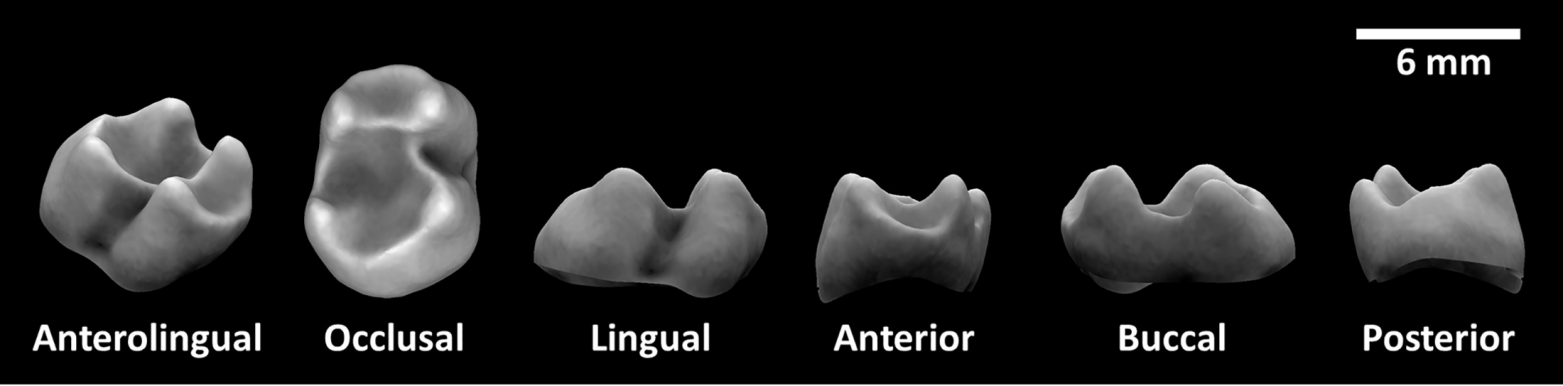
PCV performed on a M_2_ of *Alouatta seniculus* (USNM-281673, triangle count = 10,000, morphosource.org). Brighter areas of the tooth have higher PCV. Note that the tips of the cusps and edges of the crests have higher PCV values than the bottom of the basins, sides of the tooth, and along the cervical margin.

Here, we investigate a recently introduced dental topographic metric, ambient occlusion [17,23]. Ambient occlusion (herein portion de ciel visible, PCV) is a measure of how exposed a surface is to ambient lighting, and is commonly used in computer programs to make 3D objects appear more realistic. While the ambient light can shine from any direction, we are only considering light coming down from the positive z-direction, which, in the case of teeth, is the occlusal direction. Points with higher PCV can “see” more light than points with lower PCV, and are, therefore, more exposed. In terms of teeth, cusp tips, crests, and blade edges tend to be more exposed and have higher PCV values, while basins, sides of enamel caps, and enamel fissures tend to be less exposed and have lower PCV values. This is illustrated in Fig 1, where the tips of the cusps and edges of the crests of an *Alouatta seniculus* LM_2_ have higher PCV values than the bottom of the basins, sides of the tooth, and along the cervical margin.

**Fig 2:**
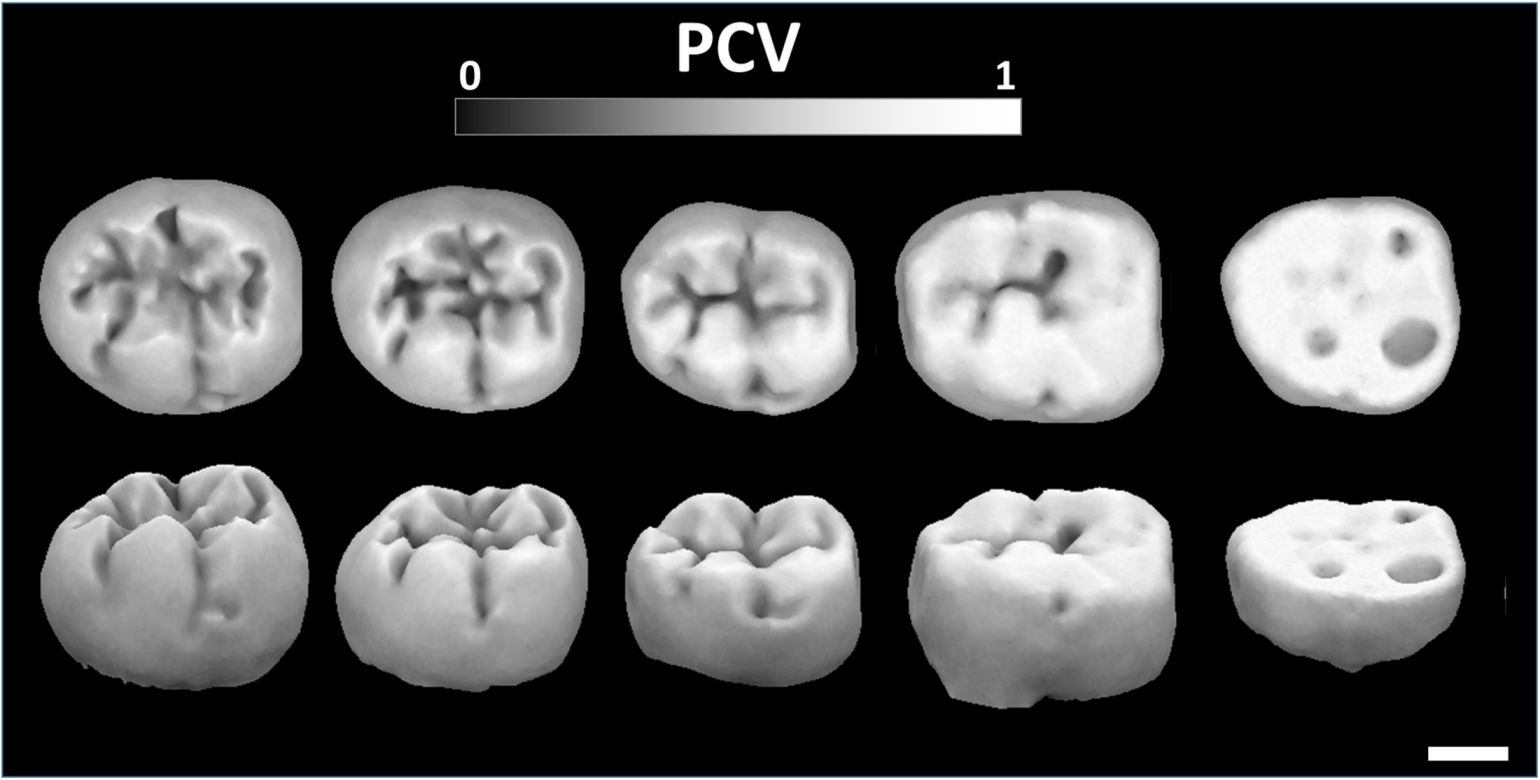
PCV contour plots on a sequence of *Paranthropus robustus* LM2s (left to right: SK25, SK55b, SK1587b, SK858, SK1586) showing increasing levels of wear (left to right, modified Scott’s wear scores: 1, 1.6, 3, 4, 5.2 [17]). All are right molars except SK1586, which was mirrored for visual comparison. PCV scale: black = 0, white = 1. Scale in bottom right corner = 5 mm. Note that when the cusps wear, the wear facets become whiter, indicating higher PCV values. Dentin pools (SK1586, right) have reduced PCV values relative to the worn dentin. The PCV values for these teeth are, from left to right, 0.547, 0.552, 0.571, 0.584, and 0.653.

Previously, PCV has been used as a metric for morphological wear resistance [17]. The logic for this argument follows that, all else being equal (i.e., chewing kinematics, food position, food mechanical properties), areas that are more exposed have a higher probability of contacting the food bolus/opposing tooth during a chewing cycle and thus have higher PCV values than areas that are less exposed. In this respect, the average PCV on a tooth can provide a gross estimate for morphological wear resistance (Fig 2). Please see the Discussion section *PCV as an indicator of morphological wear resistance* for further discussion about the correlation between PCV and morphological wear resistance and its limitations.

PCV has been used in two dental studies. The first investigated the relationship between four dental topographic variables (PCV, DNE, RFI, and OPCR) and four metrics of hard-object feeding efficiency using a set of theoretical molars [23]. It was concluded that none of the metrics tested, including PCV, was correlated to hard-object feeding efficiency. The second investigated changes in PCV between four groups of South African hominins (*Australopithecus africanus*, *Paranthropus robustus*, *Homo naledi*, and *Homo* sp.), and showed significant differences existed between taxa [17]. They concluded differences in morphological wear resistance (PCV) were likely due to an increase in intrinsic (e.g., phytoliths) or extrinsic (e.g., dust, grit) dietary abrasives.

This paper has two goals. The first goal is to quantitatively test PCV’s ability to predict diet in two groups of primates. As taller structures do a more efficient job at “shading” the tooth than shorter ones, which are, overall, more exposed to ambient light, we predict teeth with high cusps are likely to have lower average PCV values that teeth with low cusps. In primates, folivores and insectivores tend to have taller crowned M2s with higher cusps compared to omnivores, frugivores, and hard-object feeders [11,15,19,20,24,25]. As such, we predict folivores and insectivores will have lower average PCV values than omnivores, frugivores, and hard-object feeders. The second goal is to qualitatively investigate the relationship between PCV and morphological wear resistance using a cross-sectional series of worn molars.

## Materials and methods

We used a sample of 209 primate minimally worn mandibular second molars (LM_2_s), representing two groups (Prosimii, n = 111; Platyrrhini, n = 110) and five dietary categories (insectivore, folivore, omnivore, frugivore, and hard-object feeder) to test the relationship between average molar PCV and diet. The majority of this sample was used in previous dental topographic studies [11,15,18,19], and are freely available at www.morphosource.org [26]. Five teeth obtained from www.morphosource.org were not part of the aforementioned studies (see supplementary information for specimen list).

Information on the digital procurement of these data can be found in published work [11,15,20]. The same dietary categories used by [11,15,18,20] were also used here, and can be found in S1 Table, with two changes. *Galago alleni*, previously classified as an omnivore, was classified as an insectivore with the other galagos here, and *Nycticebus javanicus*, which was classified as “unknown” in [18], was classified with other *Nycticebus* in this study as an omnivore.

Downloaded teeth were imported into Geomagic Studio and rotated 180°, if necessary, so the occlusal surface was pointed in the positive z-direction. Teeth were otherwise not reoriented, as the teeth were already in standardized orientations [11], and PCV is certainly sensitive to tooth orientation. How sensitive PCV is to orientation has not been quantified and will almost certainly be affected by factors like relative tooth and cusp height, as relatively taller teeth and cusps will block more ambient light as they are rotated, but teeth/cusps with lower relief will be less affected by reorientation. Teeth were not cropped, and the entire enamel cap (EEC) was used for topographic analysis [17]. Teeth were down sampled to a target of 10,000 triangles/polygons and smoothed using the smooth surface command (lambda = 0.6, iterations = 100) in AVIZO 8.1 for comparability with other topographic studies that have used this sample [15].

### Dental topographic quantification

PCV was calculated using CloudCompare [27], an open source point cloud and mesh processing software (http://www.danielgm.net/cc/). *.ply surface files were uploaded into CloudCompare, and PCV was calculated using the “PCV” command in the “Portion of Visible Sky” (PCV, portion de ciel visible) plugin. (Note: PCV was calculated on the mesh and not the vertices.) This plugin calculates the PCV at each vertex from the positive z-direction downward using an algorithm equivalent to that found in ShadeVis (http://freshmeat.net/projects/shadevis; [28,29]). A “Sample rays on a sphere” was selected, and a default count of 256 was used with the “only northern hemisphere (+Z)” box ticked. PCV values were then averaged using the Fit a Statistical Model to Scalar Field command (Gauss distribution) to give one PCV value for the tooth. Although the density distribution of the PCV values is not normal, the Gauss distribution calculates the mean PCV value. The density distribution can be exported at this stage, should it be desired for further statistical analyses.

MorphoTester was used to calculate three other dental topographic metrics (DNE, OPCR, and RFI) to compare the strength of the relationship between PCV and diet compared to that of other published metrics [12]. 1% outlier removal was used for DNE (i.e. reporting on the bottom 99% of the Energy x area in Outlier removal)–this is equivalent to DNE99 in [17]. A minimum patch count of 3 was used for OPCR, and RFI was reported as the natural log of the square root of 3D enamel surface area divided by 2D projected area [11]. Tooth size has been shown to be correlated to diet in primates [30], and increases the ability for dental topographic measures to predict diet [15]. Although classically quantified with length and width measurements, which are analogous to 2D projected area (herein, tooth size), it can be also be quantified by enamel surface area (SA). As such, the relationship between SA, tooth size, and diet will also be investigated.

### Statistics

Statistical analyses were performed using R v3.4.2 and RStudio v1.0.136 with the package MASS [31,32]. A two-way analysis of variance (ANOVA) was run to investigate the effect of phylogenetic group and diet on topographic metrics. As the interaction between group and diet were significant for all variables except OPCR (Table 1), platyrrhines and prosimians were analysed separately.

**Table 1:** Two way ANOVA showing the effects of diet and group on topographic variables. All factors and interaction variables are significant with the exception of the OPCR interaction variable. P values of 0 are less than 0.0005.

DT metric	Factor	Df	F	P
PCV	Diet	4	70.582	0
	Group	1	99.622	0
	Diet x Group	3	12.83	0
DNE99	Diet	4	90.397	0
	Group	1	8.019	0.005
	Diet x Group	3	6.4	0
RFI	Diet	4	30.868	0
	Group	1	140.461	0
	Diet x Group	3	4.778	0.003
OPCR	Diet	4	2.888	0.023
	Group	1	36.834	0
	Diet x Group	3	0.103	0.958
Surface area	Diet	4	71.768	0
	Group	1	63.421	0
	Diet x Group	3	33.648	0
Tooth size	Diet	4	59.329	0
	Group	1	32.373	0
	Diet x Group	3	27.04	0

One-way ANOVAs with Tukey Honestly Significant Difference (HSD) pairwise comparison tests were run within each group to investigate the differences in topographic values due to diet. Linear discriminant function analyses (DFAs) were run for platyrrhines and prosimians separately and together to test the predictive ability of PCV compared to the other variables within groups and primates. The lda function, part of the MASS package in R, was used with leave-one-out cross-validation and prior probabilities of class membership set to be equal [33]. Although equal prior probabilities and unequal samples per diet group could lead to biased results, one of the uses of dental topography is to perform dietary reconstructions. During these reconstructions, researchers do not know the diets of the extinct taxa, and thus the prior probabilities would be unknown. Here, we wanted to mimic this situation.

As PCV has previously been reported to be correlated to other topographic variables [17,23], Pearson’s correlations were run between all topographic variables. Data were not separated by group, as we were interested in general correlations between variables. For all analyses, significance was determined at a value of p = 0.05 and Bonferroni corrections were used when appropriate.

### PCV and morphological wear resistance

Five *P*. *robustus* LM_2_s from [17]were chosen to investigate the relationship between PCV and morphological wear resistance. The molars represented five unique degrees of wear, ranging from essentially unworn to low to moderate dentin exposure [34]. Contour plots depicting variation of PCV values over each tooth were used to investigate the relationship between PCV values and molar wear.

## Results

**Fig 3:**
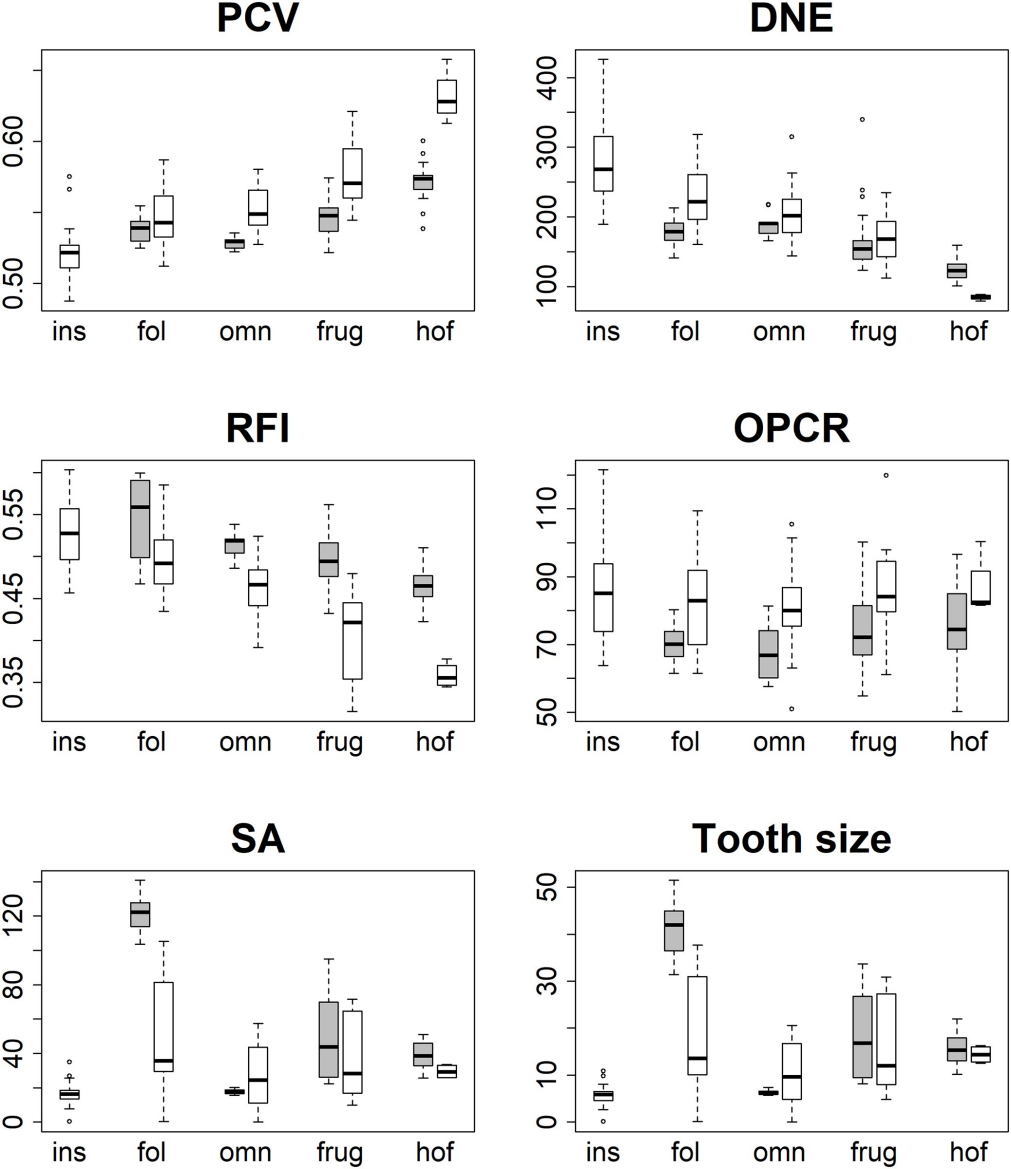
Boxplots of topographic variables for platyrrhines (grey) and prosimians (white). Ins = insectivore, fol = folivore, omn = omnivore, frug = frugivore, and hof = hard-object feeder.

Averages and standard deviations of dental topographic values for each group and dietary category are presented in Fig 3 and Table 2. Raw values are published in the supplementary material (S1 Table). PCV is generally highest for hard-object feeders, followed by frugivores, omnivores, folivores, and lowest for insectivores (S2 Table). Results from two-way ANOVAs indicated significant interactions between group and diet factors for all topographic variables except OPCR (Table 1). As such, one-way ANOVAs were used to examine the effect of diet on topographic variables in platyrrhines and prosimians separately. One-way ANOVAs revealed all topographic variables except OPCR were correlated to diet, though it should be noted that OPCR did significantly vary with diet in the two-way ANOVA (Table 3). Tukey HSD results showed differences between dietary categories occurred more often for PCV, DNE, and RFI in prosimians (Table 4), and more often for SA and tooth size in platyrrhines (Table 5). Significant correlations occurred between many of the variables, with PCV being negatively correlated to DNE and RFI, and uncorrelated to OPCR, SA, and tooth size (Table 6).

**Table 2:** Descriptive statistics of topographic metrics separated by group and diet.

	Dietary category	N	PCV		DNE		RFI		OPCR		SA		Tooth size	
			Mean	Stdev	Mean	Stdev	Mean	Stdev	Mean	Stdev	Mean	Stdev	Mean	Stdev
Platyrrhine	Ins	0	---	---	---	---	---	---	---	---	---	---	---	---
	Fol	20	0.538	0.009	177.595	19.796	0.546	0.046	69.969	5.548	121.882	10.144	41.103	5.417
	Omn	10	0.529	0.004	189.248	17.362	0.516	0.015	67.362	7.795	17.709	1.526	6.306	0.517
	Frug	40	0.546	0.012	161.239	38.12	0.498	0.029	74.963	11.660	49.425	25.158	18.118	8.826
	HoF	40	0.572	0.01	124.681	14.865	0.466	0.019	75.966	11.268	39.008	7.367	15.409	3.124
	Total	110	0.552	0.019	153.465	35.252	0.497	0.041	73.728	10.638	55.928	36.287	20.238	12.016
Prosimian	Ins	28	0.522	0.018	277.179	55.834	0.525	0.038	85.362	14.649	16.225	6.557	5.668	2.171
	Fol	28	0.545	0.019	226.721	43.763	0.498	0.040	82.513	13.129	51.874	31.269	19.174	11.436
	Omn	33	0.553	0.014	202.354	37.220	0.463	0.037	80.481	10.500	27.75	18.404	10.758	6.936
	Frug	18	0.577	0.023	167.859	36.860	0.409	0.050	85.569	12.821	39.113	23.814	16.798	9.774
	HoF	4	0.632	0.019	84.819	4.041	0.359	0.015	86.75	9.098	29.534	4.184	14.403	1.893
	Total	111	0.550	0.03	217.546	62.363	0.475	0.06	83.276	12.634	32.835	24.785	12.708	9.453

**Table 3:** Results of one-way ANOVAs, investigating for differences in mean topographic variables between diets in platyrrhines and prosimians.

	DT metric	Df	F	P
Platyrrhine	PCV	3	85.665	0
	DNE99	3	28.102	0
	RFI	3	35.847	0
	OPCR	3	2.945	0.036
	SA	3	140.887	0
	size	3	104.448	0
Prosimian	PCV	4	48.443	0
	DNE99	4	29.248	0
	RFI	4	34.909	0
	OPCR	4	0.839	0.503
	SA	4	10.734	0
	size	4	11.621	0

**Table 4:** Tukey HSD results for prosimian ANOVAs. Differences between group means are reported, followed by the adjusted p-value in parentheses. P-values of zero are <0.0005, bold cells are significant.

	PCV	DNE99	RFI	OPCR	SA	Tooth size
ins-fol	**0.024 (0)**	**50.458 (0)**	0.027 (0.1)	2.848 (0.917)	**35.649 (0)**	**13.506 (0)**
ins-omn	**0.031 (0)**	**74.826 (0)**	**0.062 (0)**	4.881 (0.565)	11.524 (0.225)	5.09 (0.106)
ins-frug	**0.055 (0)**	**109.32 (0)**	**0.116 (0)**	0.208 (1)	**22.888 (0.005)**	**11.13 (0)**
ins-hof	**0.11 (0)**	**192.36 (0)**	**0.166 (0)**	1.388 (1)	13.309 (0.769)	8.735 (0.256)
fol-omn	0.008 (0.475)	24.367 (0.201)	**0.035 (0.008)**	2.032 (0.971)	**24.125 (0)**	**8.416 (0.001)**
fol-frug	**0.032 (0)**	**58.862 (0)**	**0.089 (0)**	3.056 (0.931)	12.761 (0.281)	2.376 (0.864)
fol-hof	**0.086 (0)**	**141.902 (0)**	**0.14 (0)**	4.237 (0.971)	22.34 (0.292)	4.771 (0.8)
omn-frug	**0.024 (0)**	34.494 (0.063)	**0.054 (0)**	5.088 (0.648)	11.364 (0.367)	6.041 (0.084)
omn-hof	**0.079 (0)**	**117.535 (0)**	**0.105 (0)**	6.269 (0.883)	1.784 (1)	3.645 (0.912)
frug-hof	**0.055 (0)**	**83.04 (0.008)**	0.05 (0.161)	1.181 (1)	9.579 (0.926)	2.395 (0.983)
Significant results	9	8	8	0	3	3

**Table 5:** Tukey HSD results for prosimian ANOVAs. Differences between group means are reported, followed by the adjusted p-value in parentheses. P-values of zero are <0.0005, bold cells are significant.

	PCV	DNE99	RFI	OPCR	SA	Tooth size
fol-omn	0.009 (0.108)	11.653 (0.673)	**0.03 (0.044)**	2.606 (0.916)	**104.172 (0)**	**34.797 (0)**
fol-frug	**0.008 (0.02)**	16.356 (0.12)	**0.048 (0)**	4.994 (0.299)	**72.457 (0)**	**22.985 (0)**
fol-hof	**0.034 (0)**	**52.914 (0)**	**0.08 (0)**	5.997 (0.156)	**82.874 (0)**	**25.694 (0)**
omn-frug	**0.017 (0)**	**28.008 (0.019)**	0.018 (0.298)	7.6 (0.168)	**31.716 (0)**	**11.811 (0)**
omn-hof	**0.044 (0)**	**64.567 (0)**	**0.05 (0)**	8.603 (0.094)	**21.299 (0.002)**	**9.103 (0)**
frug-hof	**0.026 (0)**	**36.558 (0)**	**0.032 (0)**	1.003 (0.973)	**10.417 (0.028)**	2.709 (0.203)
Significant results	5	4	5	0	6	5

**Table 6:** Coefficients of correlation between topographic variables. P-values of zero are <0.0005, bold cells are significant. Significance at a Bonferroni corrected p-value of 0.01.

	PCV	DNE	RFI	OPCR	SA
DNE	**-0.701 (0)**	---	---	---	---
RFI	**-0.797 (0)**	**0.422 (0)**	---	---	---
OPCR	0.004 (0.95)	**0.483 (0)**	-0.09 (0.181)	---	---
SA	-0.091 (0.178)	-0.171 (0.011)	**0.322 (0)**	**-0.244 (0)**	---
Tooth size	0.001 (0.983)	**-0.223 (0.001)**	**0.198 (0.003)**	**-0.234 (0)**	**0.986 (0)**

Linear DFAs showed that, when only one topographic variable is used to predict diet, PCV had the highest successful classification rate in group specific analyses, and the second highest classification rate in the combined analysis (Table 7). Including size increased successful classification, and both SA and tooth size produced similar results. As the inclusion of additional variables always increases the fit of a model, the highest percent classifications occurred when PCV, DNE99, RFI, and OPCR were used together with a metric for size.

**Table 7:** Percent of successful classifications for linear DFAs using leave-one-out cross-validation and equal prior probabilities.

Variables	Platyrrhines	Prosimians	Combined
PCV	68.18	49.55	42.53
DNE99	57.27	40.54	49.77
RFI	56.36	45.95	25.79
OPCR	30	20.72	19
SA	65.45	38.74	39.82
Size	62.73	38.74	39.82
PCV*SA	90	52.25	52.49
DNE99*SA	82.73	48.65	64.25
RFI*SA	75.45	53.15	45.7
OPCR*SA	61.82	27.93	44.8
PCV*Tooth size	87.27	53.15	49.77
DNE99*Tooth size	81.82	52.25	61.54
RFI*Tooth size	74.55	51.35	44.8
OPCR*Tooth size	59.09	30.63	42.08
PCV*DNE99*RFI*OPCR	78.18	58.56	65.16
PCV*DNE99*RFI*OPCR*SA	90	71.17	73.3
PCV*DNE99*RFI*OPCR*Tooth size	90.91	69.37	74.66

In the wear series of *P*. *robustus* molars, areas of the tooth that have a higher probability of contacting a food bolus/opposing tooth during a chewing cycle have relatively higher PCV values. This is highlighted by the sections of high PCV on the expanding wear facets (Fig 2: *P*. *robustus* PCV values and surfaces from [17]). The first molar (SK25) is essentially unworn, and the highest PCV values are on the cusp tips and crests, where wear facets tend to develop. However, PCV appears to be equally high on all cusp and crest tips, and not just those that will wear first. SK55b is mostly unworn with low levels of wear on the protoconid and hypoconid, and these cusps have a higher average PCV (as indicated by the lighter coloured areas), with the areas of highest PCV corresponding with the developing wear facet. Relative to SK55b, the protoconid and hypoconid of SK1587 have higher PCV, which correspond with the expanding wear facets. Other areas of the SK1587 LM_2_ have also begun to wear and are showing larger areas of high PCV.

PCV at the wear facets was compared to the rest of the tooth by calculating PCV for the entire tooth, then individually isolating the wear facets using the “segment” tool in CloudCompare. All wear facets were merged into the same mesh (edit->merge) and average PCV was calculated for the wear facets and surrounding tooth, separately. The wear facets on this tooth have a higher average PCV value (PCV = 0.830) than the rest of the tooth (PCV = 0.575), showing these light patches do, in fact, correspond with higher PCV values.

By the time the molar has almost worn flat, but dentin has not yet been exposed (SK858), almost the entire occlusal surface of the tooth has a large patch of high PCV. Once dentin is exposed (SK1586, LLM_2_ mirrored for visualization), the enamel ridges, which serve to break foods down, have a much higher average PCV (PCV = 0.893) than the dentin pools (PCV = 0.622) and the rest of the tooth (PCV = 0.707). This is because the occlusal enamel is a nearly perfectly flat surface, with little curvature and no tall structures to obscure the ambient light, so the surface is highly exposed to ambient light.

## Discussion

Average ambient occlusion, quantified through PCV, differed significantly between dietary categories in two major primate groups, and performed as good or better than the other metrics at predicting diet from tooth shape (Tables 3 and 7). In prosimians, insectivores had the lowest average PCV values, followed by folivores, omnivores, frugivores, and hard-object feeders. In platyrrhines, omnivores had the lowest average PCV values, followed by folivores, frugivores, and hard-object feeders (Table 2).

Differences in the placement of the omnivore category between platyrrhines and prosimians could be due to the dietary classification used for *Saimiri*. Here, we used a similar sample as in [15], where *Saimiri* was categorized as an insectivore in the platyrrhine-only analyses, and an omnivore in the platyrrhine + prosimian analyses. This is because, while *Saimiri* appears insectivorous compared to the other platyrrhines sampled, it appears omnivorous compared to prosimians. Because of this, *Saimiri* may represent an intermediate morphology between an omnivore and insectivore. As it was classified as an omnivore here, this would explain why platyrrhine omnivores have a lower than expected average PCV.

Tukey HSD tests revealed that, within prosimians, more statistical differences occurred in PCV between dietary categories than any other topographic variable, indicating it performed the best at differentiating taxa based on dietary categories. There were 9 significant differences in PCV, 8 in both DNE99 and RFI, 3 in both SA and tooth size, and no significant differences in OPCR. In platyrrhines, both SA and tooth size had the highest number of differences between groups (6 and 5, respectively), followed by RFI and PCV (5), DNE99 (4), and OPCR (0). When only shape variables are considered (PCV, DNE, RFI, and OPCR), PCV tied with RFI as the most effective variable for differentiating taxa based on diet. The differing relationship between size (SA and tooth size) and diet in the two groups is interesting, and indicates metrics of M_2_ size are better indicators of diet in platyrrhines than prosimians (see also [15] for further discussion on tooth size and group-level differences in functional morphology).

The lack of significant differences in OPCR is intriguing, particularly as dietary differences in OPCR were found in platyrrhines and prosimians in [15], which used much of the same sample. The boxplots of DNE, RFI, and OPCR from Fig 2 in [15] are comparable to the boxplots of DNE, RFI, and OPCR in Fig 3 here. The same pattern of results are exhibited in the DNE and RFI boxplots, but the boxplots for OPCR are different, where larger differences exist in OPCR between dietary categories in [15]. Tukey HSD results are not comparable between studies, as [15] collated platyrrhines and prosimians into one, homogeneous, group, but they were kept separate here. However, [15] found two significant differences in average OPCR between dietary categories (folivores-hard-object feeders and omnivores-hard-object feeders), where none were found here.

**Fig 4:**
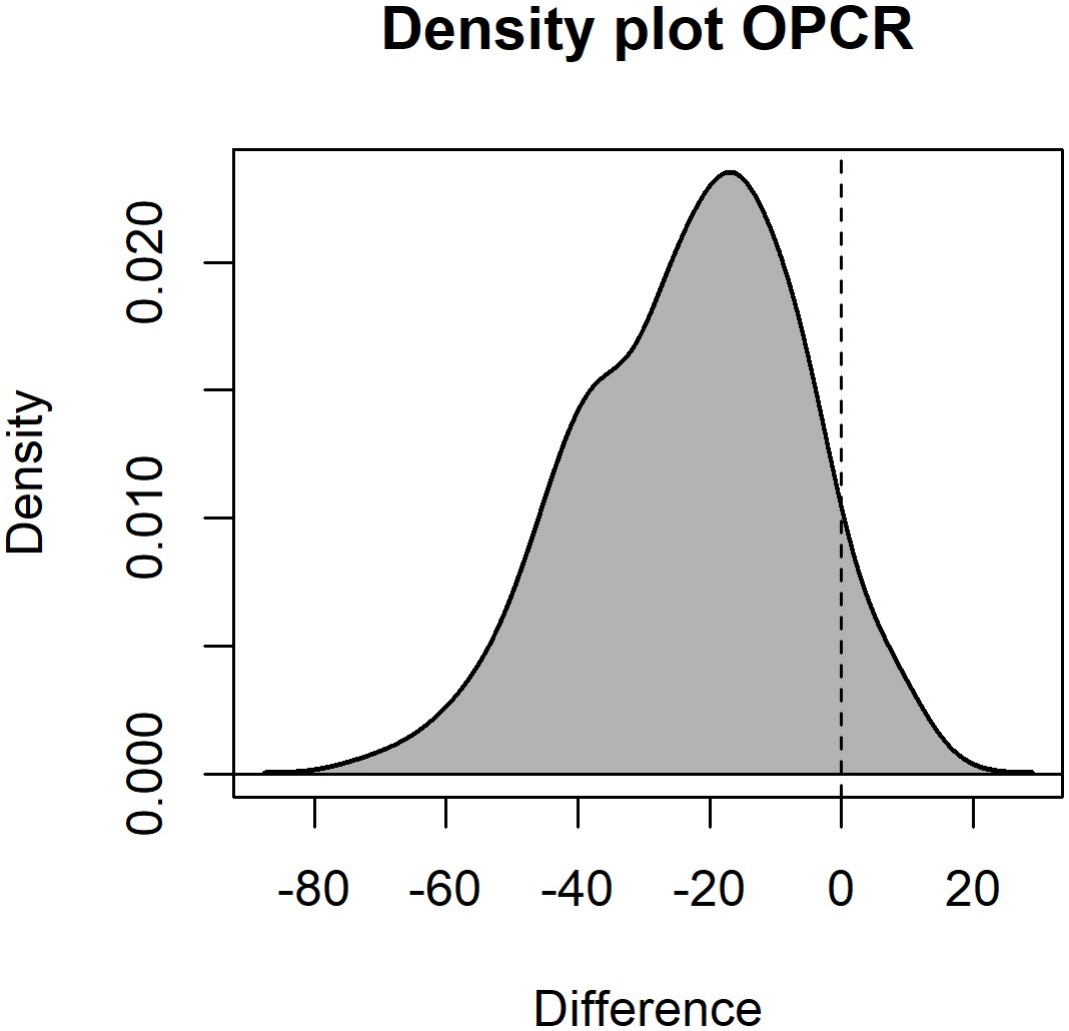
Density distributions of differences in OPCR values for a set of 216 individuals. Difference = OPCR_Winchester et al., 2014_ –OPCR_Berthaume et al., 2019_. 93.5% of the OPCR values published here are higher than in [15].

**Fig 5:**
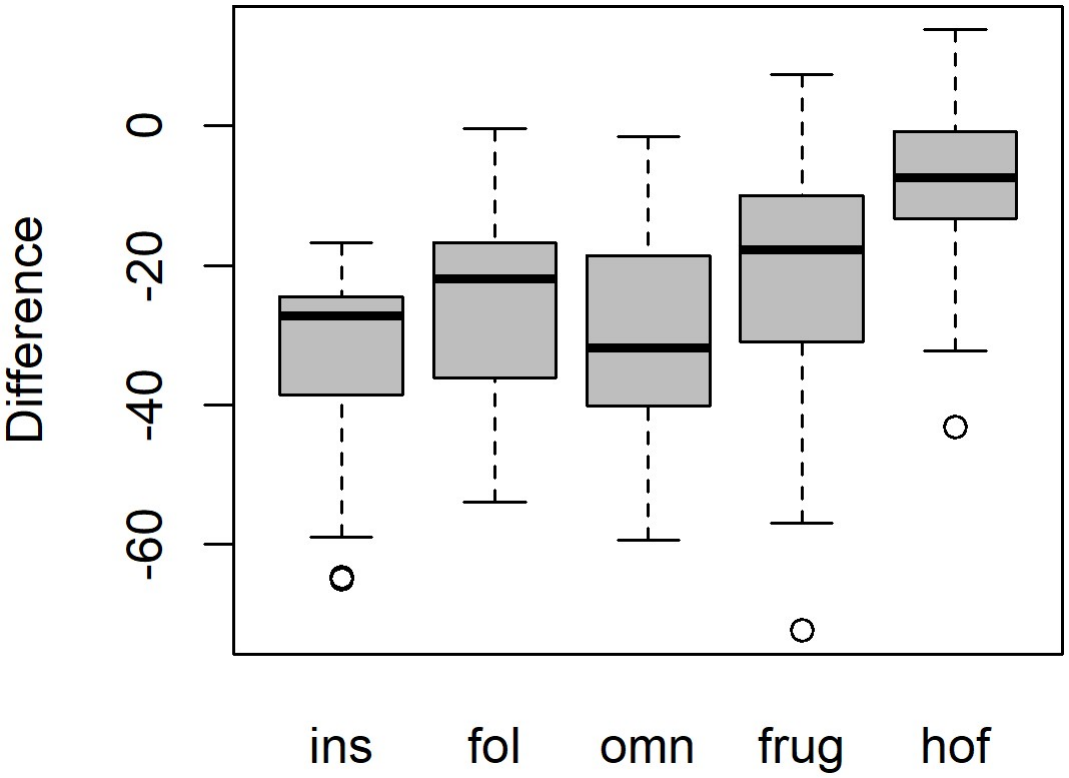
Boxplot of differences in OPCR values. Ins = insectivore, fol = folivore, omn = omnivore, frug = frugivore, and hof = hard-object feeder.

Differences in OPCR values for the 216 individuals used in both studies were investigated further. OPCR values were higher here for 202/216 (93.5%) of those individuals (Fig 4), with differences ranging from -72.375 to 13.75 (difference = OPCR_Berthaume et al. (2019)_ − OPCR_Winchester et al. (2014)_), which are large relative to the range of possible OPCR values (30.125–121.5). A one-way ANOVA revealed dietary categories were correlated to differences in OPCR values (df = 4, F = 19.3, p < 0.0005; Fig 5), with larger differences being found in insectivores and folivores than hard-object feeders and frugivores. Differences are likely due to differences in surface processing prior to OPCR calculation. [15] followed the protocol put forth by [9,35], which utilized raster-based grid data, essentially making the tooth 2.5D, and here, OPCR was calculated using a 3D polygonal mesh [12]. These results confirm the conclusions of [16,36,37], that OPCR values gathered with these two methodologies are not directly comparable.

Previously, PCV was shown to be negatively correlated to RFI but not DNE or OPCR in a set of theoretical molars [23], and negatively correlated to DNE and RFI and positively correlated to tooth size in a set of hominin M_2_s [17]. Here, PCV was negatively correlated to DNE and RFI, and uncorrelated to OPCR, SA, and tooth size (Table 6).

Linear DFAs on single topographic variables revealed PCV had the highest rate of successful classifications when platyrrhines and prosimians were separated. When platyrrhines and prosimians were combined, PCV had the second highest rate of successful classifications to DNE. Not surprisingly given the ANOVA results, OPCR performed the worst at predicting diet. When size was included, the percent of successful classifications improved greatly for all variables. The highest classification of all variables occurred in platyrrhines when PCV was combined with SA (90% successful classification). When all shape metrics (i.e., PCV, DNE, RFI, OPCR) are used, either with/without a size parameter, the percent of successful classifications ranged from 65.16–90.91%. As the percent of successful classifications is similar when just one shape metric is used with one size metric, and using all metrics risks overfitting, it appears the best model for reconstructing diet in primates consists of one shape metric (either PCV, DNE, or RFI) and one size metric.

### PCV as an indicator of morphological wear resistance

Within this wear series of molars, these probabilities of wear (i.e., PCV values) correspond well with the portions of the tooth that experience wear (Fig 2). Within unworn teeth, it does not appear to have the ability to discriminate between which cusps/crests or which portions of those cusps/crests will experience wear first, as this is a function of non-tooth shape parameters (e.g., chewing kinematics, food position, and dietary mechanical properties). For example, the tips of SK25’s cusps appear to have higher PCV values than the sides of the cusps and all cusp tips appear to have similar PCV values. However, in the more worn specimens, it becomes apparent from the developing wear facets on the sides of the cusps that the cusp sides wear more than the cusp tips, and not all cusps wear at the same time (Fig 2).

Once teeth begin to form wear facets, PCV appears proficient at providing higher values, and thereby probabilities of wear, to the wear facets. For example, the wear facets on SK1587b have higher PCV values than the surrounding areas, and cusps with large wear facets appear to have higher average PCV values than cusps with smaller wear facets. Once dentin is exposed, it provides lower probabilities to the dentin, which wears faster, than the surrounding enamel ridges. In nature, dentin wears faster than enamel, so one might expect the dentin pools to have higher PCV values than the surrounding enamel.

We believe it is acceptable for the dentin pools to have a lower average PCV when using PCV as a measure of morphological wear resistance for two reasons. First, the differences in wear rates between dentin and enamel is a function of differences in mechanical properties and not a difference in morphology, and PCV is calculated based on morphology, and not the underlying mechanical properties of the tooth. Second, PCV is being used as a probability of wear due to morphology: that is to say, it is only predicting that the shape of the enamel ridges makes them have a higher probability of wearing during a chewing cycle than the dentin pools, and this is true in nature. For a dentin pool to become deeper, the surrounding enamel must be worn away first, so the enamel ridges have a higher probability of wear than the dentin pools. If the dentin pools had a higher probability of wearing, they would become deeper at a faster rate than the enamel is wearing, and this does not occur after the dentin pools have formed. Only once the enamel is worn away does the dentin wear. One aspect of predicting wear that PCV may have trouble with is in assigning higher probabilities to dentin than enamel at the exact time dentin is exposed, before the dentin pool forms.

Two aspects of dental morphology, relative tooth and cusp height, play dominating roles in determining average PCV. In general, molar/cusp sides are more obscured from ambient light shining from the occlusal direction than the occlusal surface. Therefore, relatively taller molars and/or molars with relatively taller cusps will have a lower average PCV than relatively shorter molars and/or molars with relatively shorter cusps. This is supported by the data presented in Fig 3, where dietary categories associated with relatively taller crowned molars and relatively taller cusps (i.e., folivores and insectivores), have lower average PCV than dietary categories associated with relatively shorter crowned molars with relatively shorter cusps (i.e., frugivores and hard-object feeders). It is further supported by the negative correlation between RFI, a measure for relative molar and/or cusp height, and PCV (Table 6) [17,23].

## Conclusions

Recently, average ambient occlusion, quantified through PCV, was introduced as new dental topographic variable for quantifying morphological wear resistance, which could be used to predict dietary categories in primates. Here, we show a strong correlation between PCV and diet in two groups of primates, platyrrhines and prosimians, and that it performs as well or better than other topographical variables at predicting diet in Primates. Finally, we show how it is correlated to dental wear in a series of *P*. *robustus* teeth, and how it can be used to predict the parts of the tooth which will interact with the tooth, and, if the tooth has even low levels of wear, can be used to predict which parts of the tooth will wear further.

## Supporting information

S1 Table Raw data for analyses. Raw PCV values used for analyses.
(CSV)

S2 Table Averages and standard deviations. Descriptive statistics for PCV values.
(CSV)
